# Core–Shell Particles: From Fabrication Methods to Diverse Manipulation Techniques

**DOI:** 10.3390/mi14030497

**Published:** 2023-02-21

**Authors:** Ajeet Singh Yadav, Du Tuan Tran, Adrian J. T. Teo, Yuchen Dai, Fariba Malekpour Galogahi, Chin Hong Ooi, Nam-Trung Nguyen

**Affiliations:** 1 Queensland Micro- and Nanotechnology Centre, Griffith University, 170 Kessels Road, Nathan, QLD 4111, Australia; 2 HP-NTU Digital Manufacturing Corporate Lab, Nanyang Technological University, Singapore 637460, Singapore

**Keywords:** digital microfluidics, triggered release, sorting, assembly, targeted drug delivery

## Abstract

Core–shell particles are micro- or nanoparticles with solid, liquid, or gas cores encapsulated by protective solid shells. The unique composition of core and shell materials imparts smart properties on the particles. Core–shell particles are gaining increasing attention as tuneable and versatile carriers for pharmaceutical and biomedical applications including targeted drug delivery, controlled drug release, and biosensing. This review provides an overview of fabrication methods for core–shell particles followed by a brief discussion of their application and a detailed analysis of their manipulation including assembly, sorting, and triggered release. We compile current methodologies employed for manipulation of core–shell particles and demonstrate how existing methods of assembly and sorting micro/nanospheres can be adopted or modified for core–shell particles. Various triggered release approaches for diagnostics and drug delivery are also discussed in detail.

## 1. Introduction

Microparticles have diameters ranging from a few micrometres to a few hundred micrometres. Compared to the bulk material, microparticles have improved surface properties due to their high surface-to-volume ratio [1]. For example, higher reactivity and ease of adding functional components enable microparticles to serve as smart materials with tuneable properties. For particle sizes in the nanometre scale, surface properties become dominant compared to its core. Size-dependent optical, mechanical, electronic, and chemical properties enable nanoparticles to be used in various applications. Micro/nanoparticles have been well-researched in the past few decades and continue to be a hot topic in the fields of catalysis [2], energy [3], environment [4], pharmaceuticals [5] and biomedicine [6]. More specific applications include tissue engineering, drug delivery [7], imaging [8], and biosensors [9].

Micro/nanoparticles are categorised as homogenous micro/nanospheres or heterogeneous core–shell particles based on their material compositions. Micro/nanospheres are solid throughout, whereas core–shell particles have solid shells enwrapping cores which can be either solid, liquid, or gas [10]. The shells consist of organic polymers or inorganic materials such as metal, metal oxides, and silica or some combination of organic–inorganic materials [11,12]. On the other hand, a single material with heterogeneous structures can contain both core and shell components. The choice of shell material depends strongly on the application. These unique compositions offer features and properties that are not achievable by individual components [13,14,15]. Recently, the study of core–shell particles has been gaining attention due to emerging applications such as targeted drug delivery, biomedical science, tumour therapy, food and cosmetic industry, medicine, and material science [5,16,17,18,19,20,21]. Existing reviews focus on various strategies of fabricating core–shell particles [22], the compilation of materials that make up the shell and core components [9], and their applications [23]. A few studies also combined the topics and addressed two or more such approaches to provide more insight to readers [9,13,23]. However, a missing link remains between core–shell particle fabrication and its application. The present review attempts to bridge the gap by providing a sequential discussion, starting from the fabrication to the manipulation of core–shell particles. Additionally, a brief discussion on applications complements the review of the state-of-the-art.

Core–shell particles are fabricated by the emulsification method, where particles are produced in bulk by phase-separated emulsification or one by one in microchannels [24]. Other methods such as polymerization [25], gas shearing strategies [26], self-assembly [27], sol–gel [28], and electrospray [29] are also utilised. Knowledge of manipulating core–shell particles facilitates current applications and enables their future use. For instance, core–shell particles for drug delivery are required to effectively transport drugs to targeted sites and subsequently release them in a controlled manner [10]. To achieve this, particles need to be sorted to ensure uniform morphological properties. The release of the cargo needs to be triggered by a suitable mechanism. Thus, there is a need for an in-depth study of manipulation techniques for the effective integration of fabrication and application of core–shell particles.

**Figure 1:**
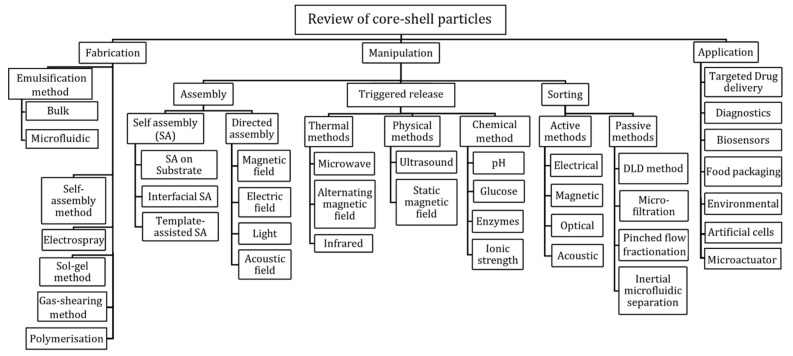
Classification of fabrication strategies, manipulation techniques, and applications of core–shell particles.

Figure 1 illustrates the key sections of this review including fabrication, manipulation, and application. Manipulation strategies are further divided into assembly, sorting, and triggered release. Assembly is a process through which building blocks arrange into an orderly manner to form a structure. The arrangement is realised either by utilising local interactions of building blocks or with the aid of external fields [30]. Although most of the reported methods on particle assembly are performed on micro/nanospheres [31], the assembly methodologies can be adopted for core–shell particles as the underlying principles relate only to their surface properties. Core–shell particles are like solid micro/nanospheres where only surface properties matter.

Sorting core–shell particles is crucial for real-world applications [32]. Particles vary in size, shape, density, and porosity due to non-ideal fabrication processes. Additionally, particles may aggregate or accumulate impurities during fabrication or storage. Existing sorting methods for micro/nanospheres need to be modified to sort core–shell particles. Size and density sorting methods can separate core–shell particles from micro/nanospheres when used sequentially [33]. On the other hand, techniques based on dielectric or optical properties can sort core–shell particles in a single stage [34].

**Figure 2:**
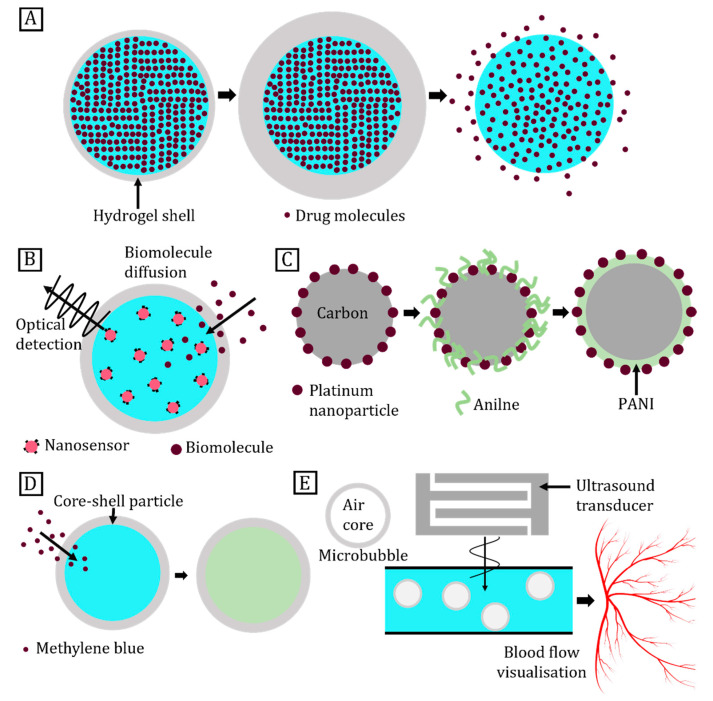
Typical applications of core–shell particles. (**A**) Chitosan core–shell particle for drug release; (**B**) Core–shell particles for biomolecule sensing and release; (**C**) Carbon-platinum-PANI (polyaniline) core–shell particles as catalysis; (**D**) Core–shell particle for methylene blue detection; (**E**) Schematics showing enhanced ultrasound imaging of blood capillaries after microbubble infusion.

Core–shell particles serve as storage and carrier platforms for many applications. This role requires the release of core materials, when triggered by external stimuli. Triggering is complete when the shell ruptures and releases the core contents. For instance, the shell can be made of biodegradable polymers for triggered release in drug delivery [35]. Typical polymers for this purpose are polyesters, polyanhydrides, polyorthoesters, polyphosphazenes, and polyurethanes [36]. Polymers can be easily tailored to respond to multiple stimuli including temperature changes [37], electromagnetic fields [38], ultrasonic waves [39], and chemical signals [40]. In addition, polymers offer the capacity to control surface and mechanical properties towards controlled degradation. The core–shell particle is a good candidate for triggered drug release due to its unique structure [16]. Figure 2A shows how the shell supports and retains the drug-loaded core until its triggered release. In some cases, drug release also couples with biosensing [41]. Figure 2B illustrates shell functionalisation to detect a given concentration of a marker that subsequently triggers drug release. Triggered release of insulin upon glucose detection is such an example [42]. The two materials enable core–shell particles to be used for catalysis [43], biosensing [5], diagnosis [17,44], water treatment [45], artificial cells [46], and encapsulation in the food or cosmetics industry [47]. Expensive metal catalysts such as platinum may take advantage of the core–shell structure to reduce cost (Figure 2C). Both the shell and core can serve as active media for catalysis. If not used as the active site, the shell serves as the protective layer, whereas the core provides support to the shell. Core–shell particles have been used for the detection of methylene blue dye (Figure 2D). Figure 2E demonstrates that core–shell particles are also employed for diagnostic purposes. Gaseous core–shell particles known as microbubbles and iron oxide core–shell particles are useful for enhancing contrast in ultrasound imaging and magnetic resonance imaging (MRI), respectively [17,44]. Hydrogel-based core–shell particles with multiple cores can serve as artificial cells [48]. Three-dimensionally cross-linked polymeric networks of hydrogel can hold up to 95% water and facilitate organelle-like partitioning, resembling natural biological processes. Such spatial confinement is highly desired for biochemical reactions [49]. Core–shell particles can overcome the limitations of conventional materials used in 3D printing. For instance, a core–shell morphology imparts enhanced mechanical and electronic properties to printed structures [50]. These are used for printed circuit boards, solar cells, transparent conductive electrodes, and touch screens [50,51]. Core–shell particles also serve as microactuators such as pump and micromixers. For instance, the liquid crystalline elastomer shell can pump core liquids across the particle shell [52]. On the other hand, magnetic core–shell particles are widely used as micromixers. The motion of the magnetic core in a rotating magnetic field enhances the sensitivity of biosensors embedded in the shell [53]. Polymer shells encapsulating metal cores are well-suited for high-energy storage applications [54]. Such conductor–insulator particles possess high electric permittivity and low dielectric loss, making them ideal for electromagnetic armour [55], piezoelectric sensors [56], and embedded capacitors [57]. Lastly, core–shell particles are used as encapsulating agents to protect the core material such as oils, vitamins, and flavours against heat, moisture, and pH changes until release [47].

## 2. Fabrication Methods of Core–Shell Particles

Fabrication methods and formulation parameters determine the physical and chemical characteristics of particles. Often, these properties are tuned to serve a specific application. Bulk emulsion coupled with solvent evaporation is a standard method for large-scale fabrication of micro/nanospheres. This method is usually combined with phase separation to obtain core–shell micro/nanoparticles [58]. However, the presence of high shear rate produces particles with a broad size distribution. Microfluidic methods are a reliable alternative for emulsification. In contrast to the bulk production with emulsion solvent evaporation, the micro/nanospheres are generated drop-by-drop with high monodispersity [5,27]. The other methods are gas-shearing, the sol–gel method, electrospray, and self-assembly. These methods create particles with varying degrees of monodispersity. Gas shearing creates core–shell microparticles with good monodispersity [26], whereas the sol–gel process yields particles with a broader size distribution [28]. Electrospraying generates monodispersed nanometre-scale core–shell particles [59]. Table 1 compares these fabrication methods in terms of particle size, dispersity and encapsulation efficiency. Although a broad range of particles can be considered as core–shell micro/nanoparticles, the fabrication process may not clearly distinguish between core–shell particles and micro/nanospheres, due to the partial coverage of the core.

### 2.1. Emulsification Method

#### 2.1.1. Bulk Emulsification

Bulk emulsification produces homogenous micro- and nanodroplets on a large scale [75]. The macroscopic droplets are mechanically broken down into uniform micro- or nanodroplets using ultrasonication or colloid milling [76]. The droplets are dried into solid spheres using solvent evaporation. Bulk emulsification also creates core–shell particles when the constituent polymers are tuned to undergo phase separation. Emulsions (i.e., water-in-oil-in-water, W/O/W; solid-in-oil-in-water, S/O/W; oil-in-oil-in-water, O/O/W) containing two polymers are employed in this process. Upon solvent removal, a thermodynamically stable configuration of phase-separated polymers is achieved. The particle size typically ranges from 1 to 800 µm [58].

The correct selection of evaporation rate and interfacial tension ensures complete coverage of the core material by the shell. Higher evaporation rates cause incomplete phase separation resulting in partial core coverage [77]. Polymers such as PLGA, Glu-PLGA, PDLLA, chitosan, and alginate are employed [78,79]. This widely used process is simple to set-up and offers a broad range of material choices. However, the low monodispersity and difficulty in scaling up remain the limitations of this process.

#### 2.1.2. Microfluidic Emulsification

Microfluidics is the science and engineering of handling fluids on the microscale. Droplet-based microfluidics has been utilised to fabricate microspheres and core–shell particles. Compared to other techniques, microfluidic emulsification offers more control over particle morphology [80,81]. The microfluidic technique allows for the emulsification of a monomer solution followed by the formation of solid microparticles. Heat or UV light exposure initiates the process of polymerisation in the presence of thermal or photo-initiators [82,83]. The size of produced particles range from 50 to 300 µm [58].

**Figure 3:**
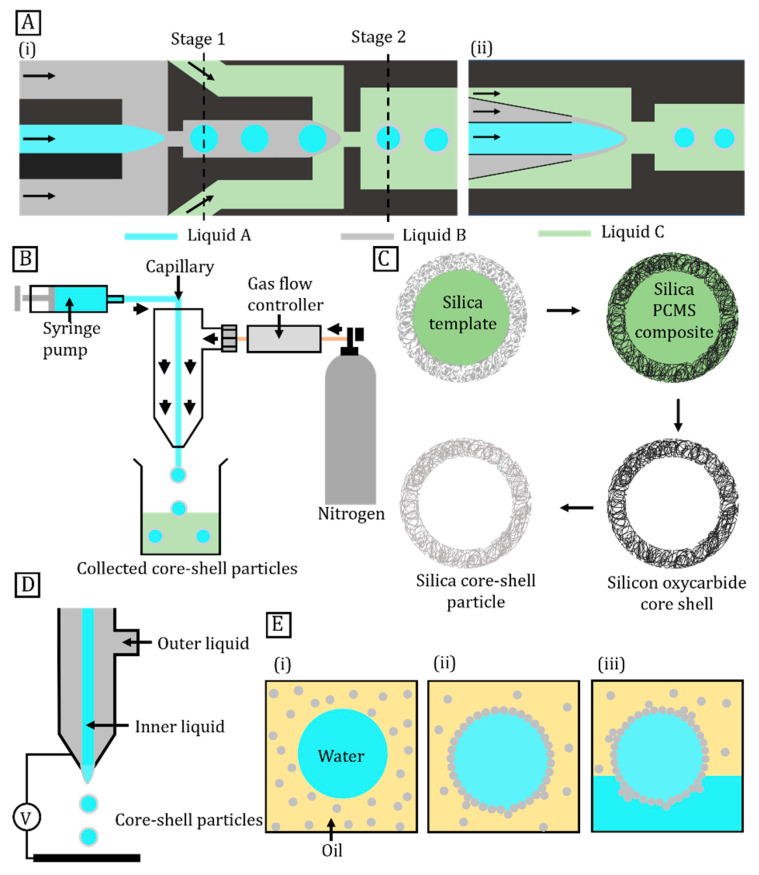
Fabrication techniques for core–shell particles. (**A**) Core–shell particle fabrication using (**i**) a two-step microfluidic method and (**ii**) a one-step method; (**B**) Experimental setup of gas-shearing method for core–shell particles; (**C**) Sol–gel method for silica core–shell particles; (**D**) Electrospray method; (**E**) Core–shell particles formation by self-assembly: (**i**) Oil-suspended water droplets and particles. (**ii**) Particles adsorb at oil–water interface to form core–shell particle. (**iii**) Transferring formed core–shell particles to water by centrifugation.

The mechanism of emulsification in microfluidic devices is straightforward. Figure 3A shows the breaking up of the dispersed phase into tiny droplets under a shearing continuous phase. The tangential drag generates a shear force on the forming droplet. This formation process can be mediated by external factors such as temperature [84,85]. Droplet size is determined by factors such as relative flow rates of the fluids; fluid properties such as viscosities and interfacial tensions as well as dimensions of the microchannel [24,86]. Sequential emulsification produces double emulsions such as the water-in-oil-in-water (W1/O/W2) type, where the oil phase separates two aqueous phases, and the oil-in-water-in-oil (O1/W/O2) type, where the aqueous phase separates the two oil phases. The resulting double emulsions subsequently undergo curing to form core–shell particles [24]. The advantages of this approach are low material consumption, minimised wastage, high monodispersity, and high core-coverage efficiency [87]. Nevertheless, this method is prone to channel contamination, blockage, and low throughputs [88]. The fabrication of double emulsions can be divided into two-step or one-step processes.

#### Two-Step Emulsification

In a two-step method, the fluid in the centre undergoes emulsification to form the droplet core (Figure 3A(i)). The droplet then disperses into a continuous phase of the outer fluid that undergoes a second emulsification. Thus, a double emulsion forms in two stages by sequentially operating two single emulsion droplet formation devices [89]. The two-step method utilises primary flow configurations such as co-flowing, T-junctions, cross-flowing, flow-focussing, and cross-flowing with opposite wettability. For example, two flow-focussing, two co-flowing, or one T-junction and one flow-focussing structures are connected in series to produce double emulsions [90]. Moreover, moving-wall geometries and microfluidic devices control the size of the single and double emulsion. Core size, shell size, and thickness are controlled by modifying the primary microfluidic structure and the flow rates [91,92].

#### One-Step Emulsification

The one-step method produces double emulsions, which subsequently are cured to form core–shell particles [93]. The microfluidic devices consist of a glass capillary inserted into a square glass channel coaxially. The size of the double emulsion is tuned by selecting channels of different cross-sectional areas [94]. Figure 3A(ii) illustrates both the core and the shell fluids flowing into the capillary channel and the outer co-axial section. The continuous phase is injected into the outer co-axial section in the opposite path. This structure creates a co-axial flow, which breaks at the outlet orifice and produces double emulsions [95].

### 2.2. Polymerisation

Micro- or nanosized core–shell particles are widely produced using polymerisation. Current polymerisation methods include emulsion, dispersion, and precipitation [25]. Emulsion polymerisation creates particles with a variety of physicochemical and colloidal properties [96]. The reaction is characterised by emulsified monomer droplets dispersed in a continuous aqueous phase and assisted by an oil-in-water surfactant [97]. The process offers flexibility as particles can be produced in a continuous, batch, or semi-batch manner [98]. Unlike emulsion polymerisation, dispersion polymerisation produces core–shell particles in two stages. First, core particles are synthesised and dispersed into the continuous phase. Next, core particles are used as nuclei in the shell formation stage. Soluble surfactants, initiators, and monomers used in this process become insoluble after polymerisation. Li et al. prepared micrometre-scale Poly(N-isopropylacrylamide) (PNIPAM)-poly(4-vinylpyridine) (P4VP) core–shell particles using the dispersion polymerisation method [99]. The major drawbacks of the process are broader size distribution and low encapsulation efficiency.

### 2.3. Gas-Shearing

Core–shell particles are fabricated by moving the core liquid through a capillary and then encapsulating the formed core in a polymer shell [26]. Figure 3B shows a simple gas-shearing apparatus for the preparation of core–shell microparticles. Controlled pulses of nitrogen gas or air interact with the core liquid at the capillary tip after passing through the annular region around the capillary. The gas applies a shear force on the core liquid stream against its surface tension to form the core. The shear force increases with the core size, opposing the surface tension. The formed core is separated from the dispersed phase at equilibrium. The outer layer of the liquid solidifies to form core–shell particles, which are later collected in a reservoir [100]. The particle size can be controlled by tuning the gas flow rate, but controlling the morphology of particles and increasing the yield are difficult [65,66].

### 2.4. Sol–Gel Method

The sol–gel process is widely employed to fabricate the core–shell structure of ceramic materials [68]. The fabrication process begins with templating colloidal particles such as nanoscale gold, silver, or cadmium sulphide particles; or microscale silica or polymer beads [22]. Next, a ceramic shell or its precursor materials are transferred onto the surface of the template particle resulting in a core–shell composite. The precipitation method, also called sol–gel condensation, completes the process. The template is subsequently removed either by calcination at high temperature or selective wet etching [28]. The sol–gel procedure starts with its impregnation of sub-micrometre-size silica particles with a preceramic polymer polycarbomethylsilane (PCMS) (Figure 3C). The pyrolysis and subsequent etching of the silica template allow mesoporous silicon oxycarbide (SOC) core–shell particles to form [101].

### 2.5. Electrospray

Electrospraying is a well-known method for fabricating polymeric core–shell particles [29]. Electrosprayed particles range from tens of nanometres to hundreds of micrometres. Figure 3D shows a typical setup that includes a high-voltage power source, coaxial needles, syringe pumps, and a grounded conductor [102]. For core–shell particles, core and shell liquids are injected into two coaxial needles using separate pumps. A voltage bias of a few kilovolts is applied between the nozzles and the grounded surface. Electrostatic forces enable coaxially flowing liquids to overcome surface tension and to break into core–shell droplets at the nozzle tip. The droplets are subsequently collected from the grounded surface [103]. The particle size ranges from 0.2 µm to 100 µm. Compared to other fabrication approaches, electrospraying yields relatively monodisperse particles. A combination of electrospraying and spray-drying methods can improve the control over particle size and morphology. Additionally, the applied electrical potential prevents the aggregation of fabricated particles [104,105].

### 2.6. Self-Assembly Method

Layer-by-layer self-assembly is a technique that encapsulates micro/nanospheres to generate core–shell particles [106]. The basis of this method is the electrostatic association between the alternate deposition of oppositely charged species. For example, multi-layered shells of polyelectrolytes, inorganic nanoparticles, or proteins form core–shell structures by sequentially accumulating on particle templates. Layer-by-layer assembly also produces organic−inorganic core–shell particles, comprising a latex core and silica shell [27,107]. Figure 3E illustrates a three-step fabrication process. First, suspension of the core liquid forms an emulsion in an immiscible liquid containing colloidal particles. Second, the emulsion droplets absorb particles to reduce the total surface energy and to cover the interface consistently. This process forms an elastic shell by locking the particles together by heating in the absence of air or by adding polycations. Third, the formed microcapsules are transferred to a solvent by centrifugation. This process eliminates the interface between the internal and external fluids for applications requiring functionalisation of colloidal particles [108,109]. Self-assembly is a quick process. However, this approach lacks specificity for the combination of core and shell materials for a broader range of core–shell particles. In addition, this method has a relatively large size distribution and low production efficiency [110].

## 3. Assembly

### 3.1. Self-Assembly

Self-assembly is a process where a collection of building blocks forms an ordered pattern without external intervention [111]. This review focuses on nano- and microscale particles as building blocks. Self-assembly is significantly affected by particle size. At the micrometre scale, most self-assembly methods utilise aqueous dispersions of a charge-stabilised spherical polymer or silica beads [112]. At the nanoscale, most methods employ surfactant-coated particles dispersed in an organic solvent [113]. Regardless of particle size, most self-assembly methods involve liquid to enable building blocks to arrange into a given configuration.

Self-assembly is driven by depletion attraction, the capillary force, dipole–dipole attraction, or their combinations in a liquid [114]. Depletion attraction arises when large particles come sufficiently close to each other to exclude small additives from their vicinities. Aggregation of blood cells upon the addition of electrolytes is an example of self-assembly. The non-uniform distribution of additives around the particles causes an osmotic pressure difference that leads to assembly. Attractive capillary interaction arises spontaneously between particles with a similar wetting nature at a fluid interface. Floating particles distort the interface and create additional surface area. Energy minimisation reduces surface distortion, causing particles to aggregate and assemble [115]. On the other hand, an evaporating solvent draws particles together while drying out through the gaps between partially assembled particles. Particles align and assemble due to capillary forces arising from the evaporating solvent. The interaction of dipole moments also facilitates particle assembly [116]. Self-assembly methods are broadly categorised into three groups, namely: template-assisted assembly, substrate assembly, and interfacial assembly. Leekumjorn et al. discussed the different self-assembly methods according to the corresponding driving forces [117].

#### 3.1.1. Template-Assisted Self-Assembly

**Figure 4:**
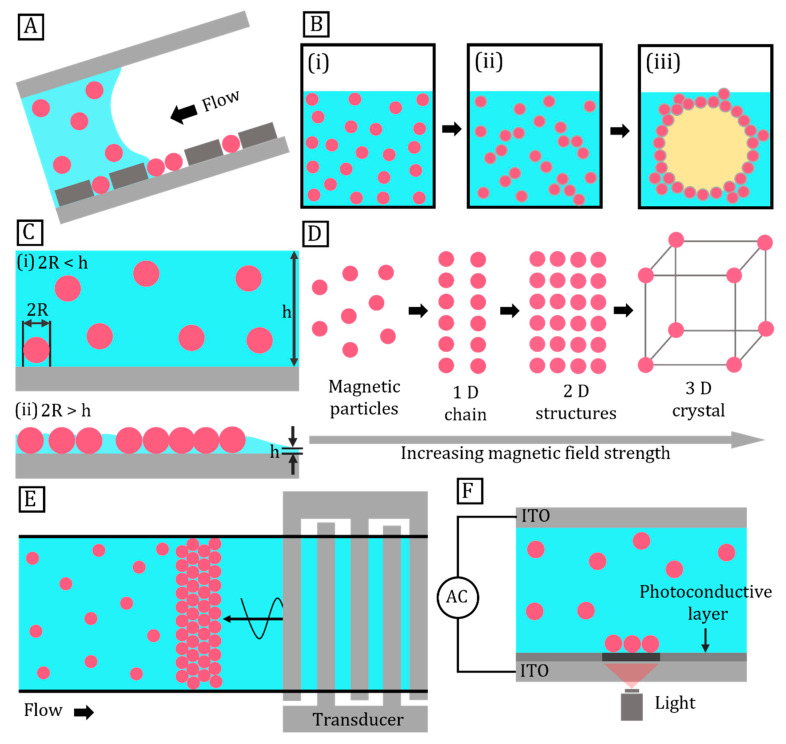
Assembly of micro- and nanoparticles. (**A**) Assembly of particles on patterned surface; (**B**) Formation of core–shell particles with layer-by-layer assembly on Pickering emulsion surfaces: (**i**) Poly (sodium styrene sulfonate) particles suspended in water, (**ii**) Surface modification, (**iii**) Emulsification to form oil-in-water Pickering emulsion; (**C**) 2D assembly of particles suspended in a medium: (**i**) Random arrangement of particles in thick layer of medium, (**ii**) Particles assembly into 2D as medium evaporates; (**D**) Suspended magnetic particles successively arrange into 1D chain, 2D sheet, and 3D crystal with increasing magnetic field strength and particle concentration; (**E**) Assembly of suspended particles in a microfluidic channel using surface acoustic wave; (**F**) Experimental setup of optofluidic assembly of particles.

Templates provide a direct method of particle assembly and patterning by introducing covalent or noncovalent interactions. Figure 4A demonstrates that topographic features of a patterned surface facilitate assembly at a similar length scale. Moreover, functionalisation of a particle surface enables controlled and selective assembly [117]. Such features enable highly localised capillary forces that guide the particles with precision over both long- and short-range orders of assembly. Particle density can be controlled by the guiding features of the template [118]. In addition to physical templates, emulsion drops serve as templating agents by confining particles into spheres suspended in a liquid. Once the particles are assembled and fixed, the droplets are dissolved, and the assembly is extracted as a colloidal suspension [119]. Figure 4B shows the layer-by-layer assembly on Pickering emulsion surfaces. This assembly uses polyelectrolytes as molecular glue to bind particles to the substrate or to particle layers. This technique produces layered particles or micropatterns on substrate surfaces [117].

#### 3.1.2. Self-Assembly on a Substrate

Substrates are used for particle self-assembly to form 1D, 2D, or 3D superstructures. In this case, surfactant-coated particles are suspended in volatile solvents such as octane, ethanol, or chloroform. The solid substrates include carbon or silicon oxide plain surfaces, or carbon-coated copper grids [120]. Figure 4C illustrates an assembly process initiated by solvent evaporation. The process creates a drying front that induces particle deposition and organisation on the substrate surface. Controlling the evaporation rate is crucial as particles either form random aggregates or close-packed assemblies depending on the evaporation rate [121,122]. For instance, latex microparticles form amorphous structures within tens of minutes of drying, whereas crystalline structures take more than a week to form. Denkev et al. reported a two-stage self-assembly on a substrate. First, the capillary force initiates nucleation which aggregates partially immersed particles [123]. Next, liquid evaporation drives the convective particle flux to grow and assemble into an ordered array. Defects may form at the drying front of the solvent due to the non-uniform drying process [124]. This defect can be minimised by allowing particles to be mobile in the interface as discussed in the next section on interfacial self-assembly.

#### 3.1.3. Interfacial Self-Assembly

Interfacial self-assembly is commonly used to place particles at the fluid–fluid interface [113]. Compared to assembly on a solid substrate, interfacial assembly is more suitable for upscaled production due to its speed and simplicity. At a fluid–fluid interface, highly mobile particles rapidly achieve equilibrium for self-assembly. This process is attributed to the rapid diffusion of particles and reagents in either fluid medium [125]. The Langmuir–Blodgett (LB) method of self-assembly is widely adopted for the liquid–air interface. Particles are confined to a 2D thin film of volatile solvent stretched over a water surface. The high surface tension of water supports the amphiphilic particles and facilitates their assembly by laterally compressing them into a monolayer [126,127].The particles are confined into a 2D thin film of volatile solvent stretched over the water surface. Liquid–liquid interfaces present in oil-in-water or water-in-oil emulsions can trap and assemble amphiphilic particles. In this case, the reduction in interfacial energy drives the self-organisation of particles and stabilises the Pickering emulsion. As assembly is achieved over a curved interface, 3D structures are formed in contrast to the planar 2D assembly with the LB method [128].

### 3.2. Directed Assembly

Directed particle assembly is driven by external factors such as light [129], magnetic field [130], electric field [131], sound [132], or their combinations [133]. The external factors control the assembly process to form large-scale ordered and hierarchical patterns [134].

#### 3.2.1. Magnetic Field

Magnetic-field-assisted assembly is unique due to its instantaneous and anisotropic interactions. This assembly method enables contactless manipulation independent of changes in temperature or pH value [135]. The strength and spatial distribution of magnetic fields can be programmed to enable fine control over the assembly [136]. Ge et al. synthesised novel superparamagnetic magnetite colloidal nanoparticles that assembled into 1D particle chains with tuneable photonic properties [137]. He et al. demonstrated that 1D photonic structures formed in a weak magnetic field exhibited a fast and reversible response to external magnetic fields [138]. Figure 4D demonstrates a step-by-step assembly of particles. Structures such as 1D chains and 2D sheets were achieved by increasing the strength and gradient of the magnetic field. This method has the potential to form a 3D assembly as well.

#### 3.2.2. Electric Field

Electrically conductive or dielectric particles assemble under an applied electric field. Particles suspended in liquid media readily respond to alternating (AC) or direct current (DC) electric fields, enabling them to be trapped or transported. Electrophoresis manipulates charged particles in a DC field, whereas dielectrophoresis manipulates dielectric particles in an AC field. Assembly is controlled by modifying the applied electric field strength and properties of the media such as dielectric constant and viscosity [131,133].

#### 3.2.3. Acoustics

Pressure nodes in standing acoustic waves have been used to trap particles for assembly. Transducers generate standing waves within a reflective microchamber and concentrate particles at the pressure nodes. However, the size of the assembly is restricted by the saturation limit of the pressure nodes [139]. An acoustics-based approach for field-induced assembly is non-specific as it interacts with a broad range of materials with different morphologies including carbon nanotubes, silver nanowires, polystyrene spheres, microscopic hydrogels, aqueous droplets in oil, and mammalian cells [140]. The directed assembly technique can be integrated with microfluidic devices to achieve continuous acoustofluidic assembly (Figure 4E) [141]. Yang et al. employed an acoustic field to tune the particle concentration and magnetic field to control interparticle interactions [142].

#### 3.2.4. Light

A temperature gradient induced by laser irradiation causes particle migration via thermophoresis. Figure 4F illustrates particle trapping on a substrate surface [143]. A low-intensity laser coupled with a patterned surface traps colloidal particles and sorts them according to the matching patterns [144]. In the case of metallic particles, 2D surface plasmon excitations at metal–dielectric interfaces direct the assembly with both optical and local thermal-convection forces [145]. Furthermore, Yamaguchi et al. assembled silica microspheres into hexagonally closely packed arrays or linear particle chains by changing the laser power [146].

## 4. Sorting

Particles are produced with a distribution of shapes and sizes and form random aggregates due to non-ideal synthesis processes. In the case of biological samples, analytes attached to microparticles may contain extraneous components. As such, the target components need to be purified or sorted subsequently [147,148].

**Figure 5:**
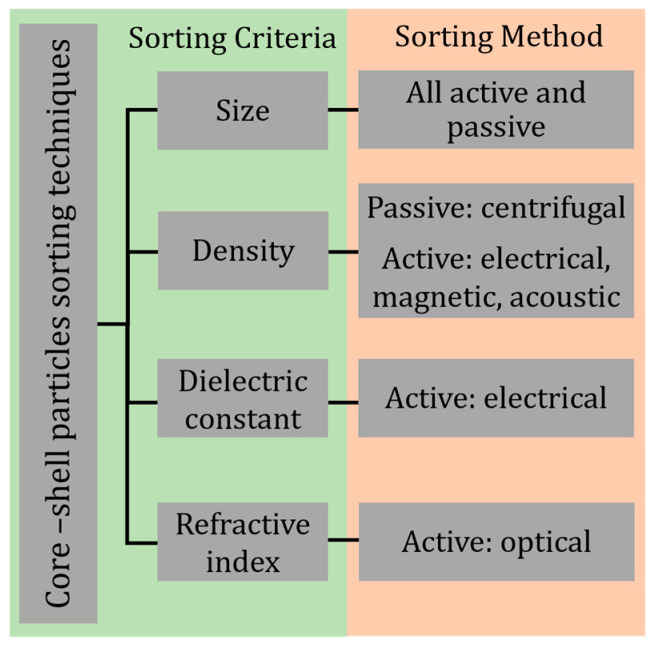
List of criteria and methods for sorting core–shell particles.

Particle sorting is critical and has application in diagnostics, chemical or biological analysis, food or chemical processing, and environmental assessment [149]. Core–shell particles introduce additional complexity to sorting as variations are present in individual core and shell components. Moreover, these particles can have ruptured shells, missing shells, or missing cores. Continuous particle sorting allows for gaining uniform and defect-free core–shell particles. This form of quality control is highly desirable for applications in chemical synthesis, mineral processing, and biological analysis [147]. Existing particle sorting strategies can be modified or directly used in sequence for sorting core–shell particles effectively. Figure 5 illustrates the key criteria and methods for sorting core–shell particles.

The sorting process involves detection and separation of particles into groups based on physical properties such as size, shape, density, deformability, electrical, magnetic, or optical properties [150]. Particle sorting techniques are broadly divided into passive or active methods. Passive methods sort particles with hydrodynamic forces without further external energy input.

Microfluidic devices sort particles passively by (i) modifying the geometry of microfluidic channels or (ii) controlling the interaction between suspended particles. The latter category includes techniques such as inertial microfluidic separation, pinched flow fractionation, microfiltration, and deterministic lateral displacement. On the other hand, active sorting methods employ external drivers such as electromagnetic, acoustic, or temperature fields. Active sorting techniques are classified based on their input energy sources [151]. Passive sorting methods do not require particles that respond to external fields. Nevertheless, active sorting methods tend to achieve higher efficiency and throughput, albeit at a higher cost due to the more complex setup.

### 4.1. Passive Sorting

#### 4.1.1. Pinched Flow Fractionation

**Figure 6:**
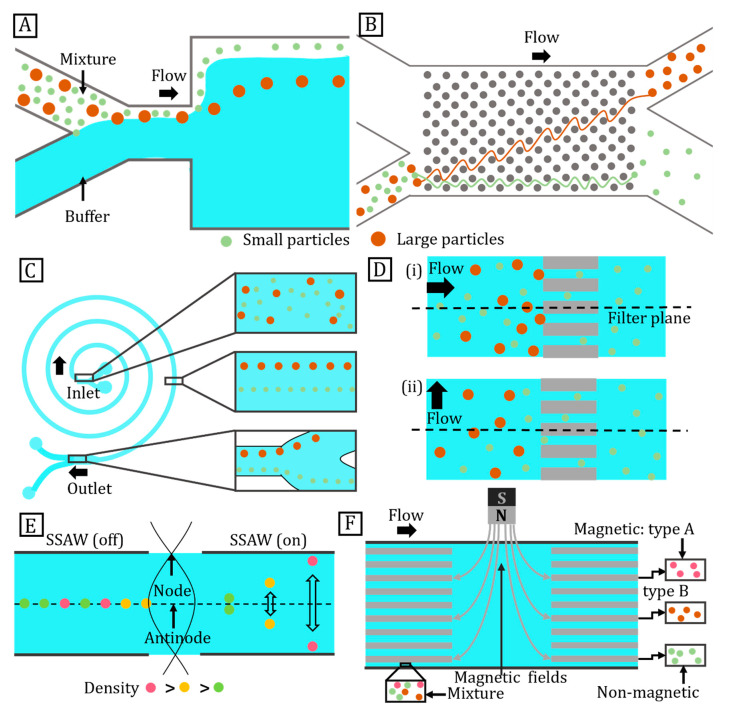
Particle sorting methods. (**A**) Microparticle sorting by pinched flow fractionation; (**B**) Microparticle sorting by deterministic lateral displacement; (**C**) Particle sorting by inertial microfluidic separation; (**D**) Schematics illustrate microfilter arrangements for particle sorting: (**i**) Dead-end filter, (**ii**) Crossflow filter; (**E**) Density-based sorting of particles in acoustic field; (**F**) Magnetic particle separation based on their magnetic properties.

Pinched flow fractionation uses laminar flow to continuously sort suspended particles based on their sizes [152]. A buffer focuses the fluid containing suspended particles (Figure 6A). In a laminar flow, the particles align with streamlines such that small particles are closer to the wall, whereas large ones are positioned away from the centre. This difference in particle position widens due to the separation of streamlines as the flow passes from a pinched segment into a broad segment. Consequently, particles are separated based on their sizes in the direction perpendicular to the fluid flow [149].

The size limit of sorted particles depends on the precise distribution of flow rates at the fluid inlets. The fractionation quality is based on the shape of the pinch and channel widening transition [147]. This technique is scalable and versatile as separation efficiency is independent of particle quantity [153]. Core–shell particles of similar size cannot be separated from solid particles using the original pinched flow technique, because of the same size. Core–shell particles can be sorted based on the difference in density between core and shell materials. Size- and density-based methods utilise pinched flow fractionation and sedimentation effects to sort core–shell particles. First, particles pass through a pinched segment for size-based sorting. Next, particles enter a vertical curved channel where the sedimentation effect separates them according to their densities [154]. Rotating the whole device introduces a centrifugal force that further enhances the sorting efficiency.

#### 4.1.2. Deterministic Lateral Displacement

Huang et al. reported particle sorting based on deterministic lateral displacement. This method continuously sorts particles with diameters ranging from sub-micrometre to several millimetres at a resolution of 10 nm. Figure 6B illustrates a specific arrangement of posts within a channel to precisely control the trajectory of particles toward separation. When a medium containing particles flows through an array of posts, the posts obstruct the flow in such a way that only smaller particles follow their defined routes. Particles are sorted relative to a critical diameter, which is set by the size of each post and the gap between them [155]. Clogging is also an issue with this method as flow stagnation in the vicinity of the posts represent unintended particle traps [149].

#### 4.1.3. Inertial Microfluidic Separation

Inertial forces drive suspended particles to stable equilibrium positions in the channel cross-section [156]. For fluid flow in a curved channel, the centrifugal force creates a secondary flow that is perpendicular to the main flow and exerts a drag force on the suspended particles [157]. Smaller particles are more susceptible to drag forces, whereas larger ones are susceptible to inertial forces. Figure 6C illustrates inertial microfluidic separation in a spiral microchannel. Ukita et al. proposed an inertial microfluidic separation method based on the density gradient of the suspended liquid suitable for sorting of core–shell particles [33]. In this method, particles are suspended in two fluids with matching densities that flow through microchannels rotating at high speeds. The channels run along the edge of a disk-shaped device such that the higher-density liquid flows on the outer side. This method can sort particles with a density mismatch as small as 50 kg/m^3^.

#### 4.1.4. Microfiltration

Microfiltration is one of the most widely used methods for separating microparticles and cells [149]. This method sorts particles according to their sizes and can be divided into three main categories according to the filtering structures: membranes, pillars, and weirs. Microfiltration utilises micropores in a membrane or gaps between pillars, serving as a sieve for sorting [158]. Membranes are especially suited for fluids with low particle concentration as they are susceptible to clogging [159]. In dead-end filters, the filter plane is parallel to the direction of fluid flow (Figure 6D(i)). Conversely, the plane of a crossflow filter is perpendicular to the fluid flow, as illustrated by Figure 6D(ii). Dead-end filtration is more efficient for capturing large particles. However, they are prone to clogging. Crossflow filtration avoids clogging as large particles are constantly washed away [160].

### 4.2. Active Method

#### 4.2.1. Acoustic Sorting

A surface acoustic wave (SAW) is an acoustic wave travelling along the surface of a substrate. A standing surface acoustic wave (SSAW) separates particles based on their physical properties such as size, density, or compressibility. Large particles experience large acoustic forces and are displaced more than smaller particles. A SSAW device is also suitable for sorting core–shell particles from solid microspheres as it allows for particle sorting based on density. The applied acoustic radiation force holds particles to pressure nodes or antinodes according to the acoustic contrast factor:

(1) $$\phi =\frac{k_{o}-k_{p}}{3k_{o}}+\frac{\rho_{p}-\rho_{0}}{2\rho_{p}+\rho_{o}}$$

where ϕ is the acoustic contrast factor, $k_{p}$ is the isothermal compressibility of the particle, $k_{o}$ is the isothermal compressibility of the fluid, $\rho_{p}$ is the density of the particle, and $\rho_{0}$ is the density of the fluid. Equation (1) shows that a SSAW has the ability to sort core–shell particles from solid microspheres based on the difference in their densities [161]. Figure 6E shows that particles denser than the suspending medium have a positive contrast factor and assemble at the nodes of the SSAW. Conversely, a negative contrast factor causes lighter particles to focus on the antinodes. As a result, denser particles migrate towards the sides of the channel, whereas lighter particles remain at the centre of the channel.

#### 4.2.2. Magnetic Field

The magnetophoretic force sorts magnetic particles in a non-uniform magnetic field according to their size and density. The migration of magnetic particles in a diamagnetic medium is termed positive magnetophoresis. The migration of diamagnetic particles in a magnetic medium is called negative magnetophoresis [162]. The magnetic force, $F_{m}$, acting on a particle suspended in a fluid is represented by:

(2) $$F_{m}=\frac{V\left(\chi -\chi_{m}\right)}{\mu_{0}}(\nabla \cdot B)B$$

where V is the particle volume, χ is the magnetic susceptibility, $\chi_{m}$ is the susceptibility of the surrounding medium, $\mu_{0}$ is the magnetic permeability of air, and ∇⋅B represents the field gradient of the magnetic flux density B. Sorting based on the magnetic field is simple and can be applied to particles having various magnetic behaviours. Conversely, density-based sorting methods are only applicable to diamagnetic particles and require a complex magnetic levitation setup. The simplest approach is sorting particles based on their differences in magnetization [163]. A magnetic field perpendicular to a fluid flow deflects magnetic particles and diverts them into distinct collection zones (Figure 6F). Lower flow rates ensure efficient separation and prevent inertial forces from dominating magnetophoresis. The magnetophoretic force experienced by the particles depends on its size and magnetic characteristics. Large particles deflect more than smaller ones. Magnetic levitation can also be used for particle sorting. A typical magnetic levitation platform consists of two magnets with the same poles facing each other, forming a non-uniform magnetic field between them. Particles of different densities levitate at distinct heights depending on the balance between buoyancy force and magnetic force [164]. This method was reported to sort particles with a density difference of 60 kg/m^3^ [165].

#### 4.2.3. Electric Field

Dielectric particles polarise and experience a dielectrophoretic force in a non-uniform electric field. This force enables suspended particles to migrate in a liquid. The method is especially useful when the particles and the medium have significantly different dielectric properties. For example, polymeric particles are much less polarisable as compared to the surrounding liquid such as water. The resulting negative dielectrophoretic effect was utilised in a curved microchannel to sort particles based on their sizes [166].

As dielectrophoretic force is directly proportional to particle size, large particles are deflected from the streamlines. Equation (3) describes the relationship between dielectrophoretic velocity $U_{DEP}$ and the applied electric field, whereas (4) indicates the dependence of the dielectrophoretic mobility of particles on its size and properties of the medium:

(3) $$U_{DEP}=\mu_{DEP}(E\cdot \nabla E)$$

(4) $$\mu_{DEP}=\frac{\varepsilon_{f}d^{2}\sigma_{p}-\sigma_{f}}{6\eta_{f}\sigma_{p}+{2\sigma }_{f}}$$

where $\mu_{DEP}$ is the dielectrophoretic mobility of particles, E is the local electric field, *ε_f_* is the permittivity of the fluid, d is the particle diameter, $\eta_{f}$ is the fluid viscosity, $\sigma_{p}$ is the electric conductivity of the particles, and $\sigma_{f}$ is the electric conductivity of the fluid [167].

Core–shell particles for biological applications have a polymeric shell and an aqueous core. The mismatch in dielectric properties allows for sorting of the core–shell particles [168]. In the case of an AC electric field, the dielectrophoretic force depends on the field strength, AC frequency, and properties of the medium. The applied electric field generates positive or negative dielectrophoretic responses from the particles depending on the frequency. Dielectrophoretic sorting is achieved when the particles move towards the local maxima or minima of the electric field [169].

#### 4.2.4. Light

An optical tweezer is a tightly focused laser beam that traps particles [170]. Particles are trapped in directions perpendicular to and along the beam propagation axis. The trapping capability depends on the difference in refractive index between the medium and the trapped particles, the size and mass of the particles, the laser wavelength, and the absorption rate of the particles [171,172]. Optical tweezing is commonly used to separate particles according to their size, density, and refractive index. Exposing two particles of the same size but different refractive indices to changing wavelengths of light, Zhang et al. demonstrated that the optical force changes faster for the particle with a higher refractive index, enabling their separation [34].

## 5. Triggered Release

Core–shell particles serve as biological sensors [5], cell culture platforms [18], and image contrast enhancing agents in diagnostics [173]. These particles are also widely used in storage, transport, and triggered release of drugs [13]. These applications require the particle shell to isolate the core from its surrounding to avoid contamination. The shell should physically support the core without unintended release. Keeping these constraints in mind, the shells are designed to respond to a specific stimulus in one or more stages [39]. For instance, PNIPAM incorporated with gold nanoparticles is a common heat-responsive polymer that expands or contracts when exposed to infrared (IR) irradiation [174].

**Figure 7:**
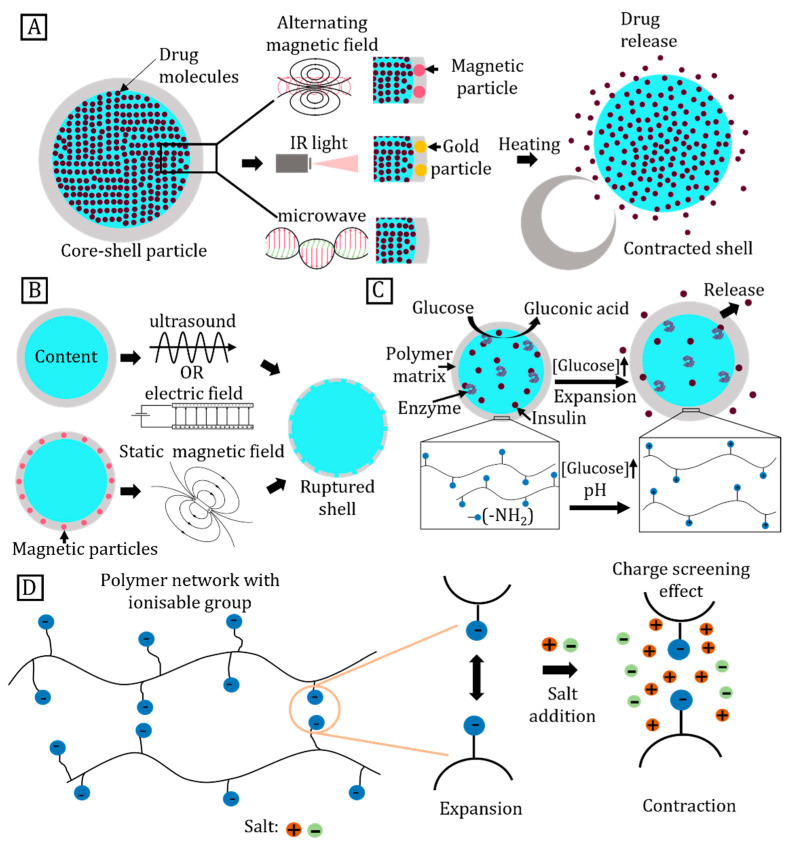
Triggered release of core−shell particles. (**A**) Schematics of triggered release of core−shell particles using various heating strategies; (**B**) Physical rupturing of shell; (**C**) Core−shell particles encapsulating insulin and enzyme, enzymatic action on glucose coverts it into gluconic acid, high pH causes shell expansion and triggers release; (**D**) Ionic concentration tunes electrostatic forces to control the expansion of the shell.

Triggered release of core–shell particles occurs when external stimuli cause significant changes in material properties. As a result, the shell releases the core content into the surroundings. Triggered release strategies include thermal, physical, and chemical methods [175]. Thermal methods utilise temperature-dependent changes in the properties of shell materials [37]. Figure 7A illustrates various heating strategies for triggered release. Electromagnetic fields in the form of an alternating magnetic field, microwaves, or IR irradiation directly heat the core–shell particles. On the other hand, indirect heating targets the suspending liquid instead of the core–shell particles. Physical methods rely on mechanically rupturing the shell via introduction of microbubbles or magnetic particles. Figure 7B illustrates common methods for rupturing the shell. Chemical methods are suitable for biocompatible materials that respond to stimuli such as pH, glucose concentration, ionic strength, and enzymatic catalysts.

### 5.1. Thermally Triggered Release

Core–shell particles with a heat-sensitive polymeric shell respond to changing temperature either by expansion or contraction, and the shell expands to form a loose polymer network that leads to triggered release [41]. Such a volumetric phase transition of particles is based on the degree of cross-linking of the polymeric network. A temperature-sensitive polymer is made up of monomers such as N-isopropylacrylamide (NIPAM) [176], N,N-diethylacrylamide (DEAM) [177], methylvinylether (MVE) [178], and N-vinylcaprolactam (NVCl) [179]. Temperature-sensitive polymers are categorised as: (i) Positive temperature-sensitive polymers that expand at high temperature and contract upon cooling below the upper critical solution temperature (UCST); or (ii) negative temperature-sensitive polymers that expand at low temperatures and contract on heating above the lower critical solution temperature (LCST) [180]. This substantial change in volume around a critical temperature is useful for controlled drug delivery [181].

#### 5.1.1. Heating by an Alternating Magnetic Field

Core–shell particles can generate heat for triggered release using nanometre-sized magnetic particles embedded in the shell. In the presence of an alternating magnetic field, magnetic moments force the magnetic particles to align with the field. At high frequencies, the magnetic moment lags the inducting field. Hysteresis between the magnetising alternating field and the induced magnetic moment results in energy loss as heat [182]. In addition, magnetic particles suspended in the core liquid also heat the core in the presence of alternating magnetic fields [183].

The amount of dissipated heat can be determined from the enclosed area of the magnetisation (*M*)–magnetising field (*H*) cycle [184]. The heating efficiency of the magnetic particles is evaluated by its specific heat absorption rate (SAR):

(5) $$SAR=\frac{f}{\rho_{MNP}}\mu_{0}\oint M(H)dH$$

where f is the frequency of oscillation, $\rho_{MNP}$ is the density of the magnetic material, and $\mu_{0}$ is the permeability of free space [185].

#### 5.1.2. Microwave Heating

Polar molecules serve as electric dipoles due to the imbalance in charge distribution. The electric field of the microwave creates a torque on the electric dipole which rotates and aligns itself with the alternating field [175]. At a microwave frequency of 2.45 GHz, a time delay exists between the frequency of the alternating field and the rotating motion of electric dipoles. This delay corresponds to the loss of the electromagnetic energy in the form of heat, similar to heating induced by a magnetic field [186]. Microwave heating depends on material properties such as electric conductivity, permittivity, and permeability as well as irradiation conditions such as electromagnetic field intensity and frequency. Microwave irradiation causes three types of heating, namely: (i) conduction loss heating, (ii) dielectric heating, and (iii) magnetic loss heating. Accordingly, the thermal energy *P* produced per unit volume is shown as:

(6) $$P=\frac{1}{2}\sigma {\left|E\right|}^{2}+\pi f\varepsilon_{0}\varepsilon_{r}^{"}{\left|E\right|}^{2}+\pi f\mu_{0}\mu_{r}^{"}{\left|H\right|}^{2}$$

where $\left|E\right|$ and $\left|H\right|$ denote the electric and magnetic field strength of the microwave, respectively; f is the frequency of the microwave; $\varepsilon_{0}$ is the dielectric constant in vacuum; $\varepsilon_{r}^{\prime \prime }$ is the relative dielectric loss factor; $\mu_{0}$ is the magnetic permeability of vacuum; $\mu_{r}^{\prime \prime }$ is the relative magnetic loss [187].

#### 5.1.3. Infrared Heating

Infrared (IR) light interacts with metal nanoparticles such as gold and produces heat via the photothermal effect. The nanostructures lead to a significant localised temperature rise [188]. When irradiated with a given wavelength, free electrons in metals collectively oscillate in phase with the electric field of the incident light and creates surface plasmon resonance (SPR). This resonance increases the strength of the electromagnetic field at the metal surface by multiple folds [189]. The energy stored in the form of surface plasmons is released via re-emission of light or dissipation of heat. When heat dissipation is the dominant mechanism for energy release, it substantially increases the temperature of the metal particles and their immediate surroundings [181].

### 5.2. Physically Triggered Release

#### 5.2.1. Ultrasound

Ultrasound is a longitudinal pressure wave at frequencies greater than 20 kHz [181]. As they propagate through a medium, ultrasound waves attenuate due to absorption and scattering acoustic energy. This attenuation increases with frequency and results in pressure variation, heating, and cavitation. At frequencies above 1 MHz, ultrasound ruptures thermosensitive core–shell particles via localised heating [190].

The cavitation effect is used to trigger the release of another category of core–shell particles known as microbubbles that consist of micrometre-sized bubbles encapsulated with surfactants, lipids, proteins, polymers, or a combination of these materials [17]. The high compressibility of microbubbles enables them to deform throughout the compression and rarefaction cycles of ultrasonic waves. The type of cavitation depends on the amplitude and frequency of the ultrasound wave as well as the size and material properties of the microbubble. Cavitation is categorised according to the mechanical index (MI), which is determined by the peak negative pressure (PnP) and the centre frequency ($F_{c}$) as:

(7) $$MI=\frac{PnP}{\sqrt{F_{c}}}$$

Microbubbles exist either in a stable regime or inertial cavitation regime, depending on their MI. The inertial cavitation regime appears at MI≥0.8, where shock waves generated at lower frequencies and higher-pressure conditions can be used for triggered release. At MI≤0.8, microbubbles undergo stable cavitation over many acoustic cycles and find applications as contrast enhancers in diagnostic imaging [191].

#### 5.2.2. Magnetic Field

The magnetic force $F_{m}$ acting on a magnetisable particle of volume $V_{p}$ and magnetic susceptibility $\kappa_{p}$ placed in a magnetic field *H* and medium with susceptibility $\kappa_{f}$ is [184]:

(8) $$F_{m}=\frac{1}{2}\mu_{0}(\kappa_{p}-\kappa_{f})V_{p}\nabla H^{2}$$

where $\mu_{0}$ is the magnetic permeability of vacuum. When such particles are embedded into a polymeric core–shell particle and exposed to an alternating magnetic field, the magnetic and polymeric shell periodically deforms. The oscillatory motion physically ruptures the shell and pumps the core material into the surroundings [43]. Low frequency minimises heating caused by the alternating magnetic field on the core–shell particle [184].

### 5.3. Chemically Triggered Release

#### 5.3.1. pH

Structures made of pH-sensitive polymers rupture when placed in an acidic or basic medium due to polymer dissociation [192]. Utilising these properties, the shell of core–shell particles can be triggered to release its content by changing the surrounding pH. Polyacids such as poly(acrylic acid) (PAA) or poly(methacrylic acid) (PMAA) contain carboxyl groups that dissociate in basic pH environments, leaving negatively charged COO-groups within the matrix that repel each other. The elongated polymer chains increase the pore size of the shell which allows the surrounding water to expand the polymer [193]. The opposite effect occurs in polybases such as poly(N,N’-dimethylamino ethyl methacrylate) (PDMAEMA), which accept protons from an acidic environment. The pH-change-triggered transition between the expanded and contracted state of the polymer network generates a pumping effect and forces the core to diffuse out of the matrix. Pore size expansion of pH-responsive polymers is controlled by combining different proportions of polyacid/base monomers in the copolymer matrix, or by modulating the electrostatic repulsion between charged species [41].

#### 5.3.2. Glucose

Core–shell particles were used to transport insulin to glucose-rich environments within the human body for diabetes treatment [41]. The insulin-loaded core is encapsulated by a pH- and glucose-sensitive hydrogel shell. The polymer matrix of the shell incorporates glucose oxidase (GO_x_) that catalyses glucose to gluconic acid. Such core–shell particles are sensitive to low glucose concentrations due to the high effectiveness of enzyme catalysis. The resultant gluconic acid dissociates and reduces the pH of the medium, thus causing triggered release. Figure 7C illustrates the pH-sensitive core–shell particle releasing insulin into the surrounding environment [42]. GO_x_ was also used in conjunction with polybases such as poly(2-hydroxyethyl methacrylate-co-N,N-dimethylaminoethyl methacrylate) (poly(HEMA-co-DMAEMA)) [194]. Polybases expand in low-pH environments to achieve the same effect of insulin release [195].

#### 5.3.3. Enzyme-Responsive Materials

The high specificity of enzymatic catalysts is highly desirable for biological applications [196]. Enzyme-responsive materials are typically supported on substrates and polymer components that control interactions to achieve macroscopic changes [197]. These changes include expansion of the polymer matrix, solubility variation, or transformation of surface properties that lead to changes in pore size [195]. These materials respond to a range of enzymes including lipases, proteases, phosphatases, and redox enzymes. Enzyme-responsive materials differ according to their hydrogel structures such as: (i) materials that undergo the sol–gel transition after enzymes selectively hydrolyse crosslinked structures; or (ii) materials with crosslinks that carry freely hanging enzyme-sensitive components. Expansion or contraction occurs with an intact overall crosslinked structure [198]. Additionally, non-polymeric enzyme-supporting substrates respond to an external stimulus by changing hydrophobicity or adhesion capabilities [196].

#### 5.3.4. Ionic Strength

The ionic strength of a medium affects the size of the micelles, solubility, or expansion of the polymeric networks for a polymeric shell [199]. For metallic shells, surface cavitation that weakens the shell occurs in the presence of salt solution of highly reactive metals [200]. Ionic solution causes polymeric shells to deform to the point of disintegration [201]. This mechanism has been applied for triggered drug release in an ionic environment [202]. Expansion and contraction characteristics depend on charged groups attached to the shell. Figure 7D shows that variation in the ionic concentration of the surrounding medium shields the charged group and prevents shell expansion. For example, in a low-ionic-strength environment, the shell expands more due to the repulsion between charged groups. Conversely, higher ionic strength screens the repulsion and limits shell expansion.

## 6. Conclusions

The present paper discusses manipulation strategies of core–shell particles according to assembly, sorting, and triggered release. The paper also includes fabrication strategies and applications of core–shell particles. Core–shell particles offer applications that are not achievable with individual core and shell components. Core–shell particles find applications in drug delivery, biosensing, diagnostics, food packaging, catalysis, 3D printing, microactuators, and water treatment due to their stability, protection against contamination, and on-demand triggered release.

Core–shell particles utilise well-studied assembly techniques of micro/nanospheres that harness surface properties of the particles. These methods are categorised as self or directed assembly based on their sources of energy. Particle sorting is a principal method that ensures quality control during the fabrication process. This review also discusses how existing sorting techniques are modified to separate core–shell particles based on size or density. Lastly, we reviewed thermal, physical, and chemical methods for triggered release of the core.

Large-scale production of uniform core–shell particles is challenging due to limitations associated with their fabrication process. For example, core–shell particles produced by emulsification or sol–gel have limited application due to the use of a surfactant and inhomogeneous particle surface. Electrospray requires a high voltage that restricts types of suitable liquids for particle production. Microfluidic methods combined with electro-hydrodynamics can create highly monodisperse particles. Nevertheless, a combination of multiple methods may overcome limitations of the individual techniques. Manipulating core–shell particles also remains a challenging endeavour due to the complexity of adapting established methods for conventional micro/nanoparticles to core–shell particles. Sorting particles using microfluidics usually results in low throughputs. Furthermore, microfluidic devices are prone to blockage, which increases the operational cost and turnover time. A combination of active and passive methods can increase sorting efficiency. However, this hybrid approach needs to address the throughput bottleneck created by the least productive component within the sorting system. In terms of triggered release of core–shell particles, the general research direction inclines towards multi-responsive materials. Candidate materials include polymers that merge the LCST or UCST phase transition with another response such as pH. Such multi-responsive materials will broaden the application of core–shell particles as sensors or drug delivery vehicles. Despite their promising potential in drug delivery and bioimaging, the design and implementation of core–shell microparticles remain difficult due to the easy degradation of multi-responsive materials inside the human body and require further extensive studies.

## Figures and Tables

**Table 1:** Comparison of various fabrication methods for core–shell particles.

Fabrication Method	Materials: Shell/Core	Particle Size (µm)	Dispersity	Encapsulation Efficiency
Emulsification	poly (DL-lactide-co-glycolide) (PLGA)/Aqueous media [60] Poly (DL-lactide) (PDLLA)/Dichloromethane [58] Poly(L-lactide) (PLLA)/PLGA [61] Alginate/PLGA [62]	45–350	Monodisperse (coefficient of variation, COV < 10%)	High (90–100%)
Polymerisation	St/Methyl methacrylate (MMA) [63] γ-methacryloxypropyltrimethoxysilane (MPS)/Polystyrene [25] St/Silica [64]	0.2–10	Polydisperse (COV = 20–50%)	High (>80%)
Gas-shearing	Chitosan/Aqueous media [65] Chitosan/Alginate [66]	20–250	Monodisperse (COV < 8%)	Low (30–70%)
Sol–gel	SiO_2_/ZnO [67] TiO_2_/Air [68] SiO_2_/Au [69]	0.1–0.4	Polydisperse (COV 40–60%)	High (80–100%)
Electrospray	Polycaprolactone (PCL)/Sudan red [70] Alginate/PLGA [71] Chitosan/PLGA [72]	0.2–100	Polydisperse (COV = 5–40%)	High (65–100%)
Self-assembly	Polymethylmethacrylate (PMMA) particle/Water [73] Latex particles/Oil [74] Polystyrene particles/Oil [73]	800–5000	Polydisperse (COV = 40–70%)	Low (<50%)

## Data Availability

No new data were created or analysed in this study. Data sharing is not applicable to this article.
